# Congenital Zika syndrome phenotype in a child born in Brazil in December 2011

**DOI:** 10.1002/ccr3.1810

**Published:** 2018-09-12

**Authors:** Katia E. F. A. Coelho, Grasiele L. C. C. Silva, Suely F. Pinho, Alessandra L. de Carvalho, Cristian M. Petter, Ivar V. Brandi

**Affiliations:** ^1^ Genetics Department Hospital Sarah Salvador Salvador Bahia Brazil; ^2^ Pediatrics Department Hospital Sarah Salvador Salvador Bahia Brazil; ^3^ Neurology Department Hospital Sarah Salvador Salvador Bahia Brazil

**Keywords:** congenital Zika syndrome, dengue, fetal brain disruption sequence, isochromosome X, microcephaly, Zika virus

## Abstract

We report a case of a Brazilian child born in 2011 with congenital Zika syndrome phenotype. Zika virus (ZIKV) may have been circulating in Brazil more than 4 years before the outbreak. ZIKV infection might be considered in children with this phenotype even without known circulation of ZIKV.

## 1 INTRODUCTION

Zika virus (ZIKV) is a mosquito‐borne flavivirus, a member of the family Flaviviridae. It is transmitted by *Aedes aegypti* and was first isolated in 1947 from rhesus monkeys in the Zika forest of Uganda.

There were no cases of human infection until 1954. Before 2007, only sporadic cases of human infection were reported in Africa and Asia. Surprisingly, an outbreak was identified in the Yap Island, Micronesia in 2007.^1^ Multiple cases were reported in Thailand between March 2012 and July 2014. Single cases were confirmed in the Philippines and in travelers who visited Indonesia in 2012.^2^ Between 2013 and 2014, ZIKV was responsible for a large epidemic in French Polynesia with a geographic expansion of the Asian lineage.^1^ ZIKV autochthonous transmission was reported in Northeastern Brazil in May 2015. The patients manifested symptoms resembling dengue virus (DENV) infection. Brazil is the country most affected by ZIKV‐positive cases.^1^

To date, Brazil is the only country to report both a large outbreak of ZIKV and newborns with microcephaly. A possible association between ZIKV infection in pregnancy and fetal malformations was raised by a local medical community in October 2015.^1^ At that time, Zika infections had already peaked in many parts of the country. Its rapid spread across this population that is probably due to the effectiveness of its vector, the *Aedes aegypti* mosquito.

## 2 CLINICAL REPORT

We report a case of a 5‐year‐old Brazilian boy born in December 2011 in Feira de Santana‐ Bahia, with severe microcephaly, clinical, and radiologic signs of Zika virus infection.

The mother got pregnant at 31 years. During the second month of pregnancy, she had a fever, headache, myalgia, rash, and signs of dengue infection confirmed by serologic tests. During the fifth month of gestation, fetal microcephaly was detected by ultrasound examination. The mother (gravida 1, para 1) had negative serology for HIV, HTLV, hepatitis C virus, syphilis, toxoplasmosis, rubella virus, and cytomegalovirus. The parents and close contacts of the family did not travel outside of Brazil prior or during index pregnancy.

The boy was the only child of the couple. The father was 42 years old. There was no consanguinity between the parents.

The child was born by Cesarean section during the 40th week of gestation. His birth weight was 3.275 g, length 47 cm, and head circumference 30.5 cm (−3 SD). Apgar scores were 9 and 10. Echocardiography revealed interatrial communication of 3.4 mm. Fundoscopy was normal. Retinal mapping revealed retinal pigment epithelium pigmentary rarefaction. Cerebrospinal fluid (CSF) examination on the second day of life had an elevated total protein (89 mg/dL) with a normal glucose level (35 mg/dL) and normal white blood cell count.

**Figure 1:**
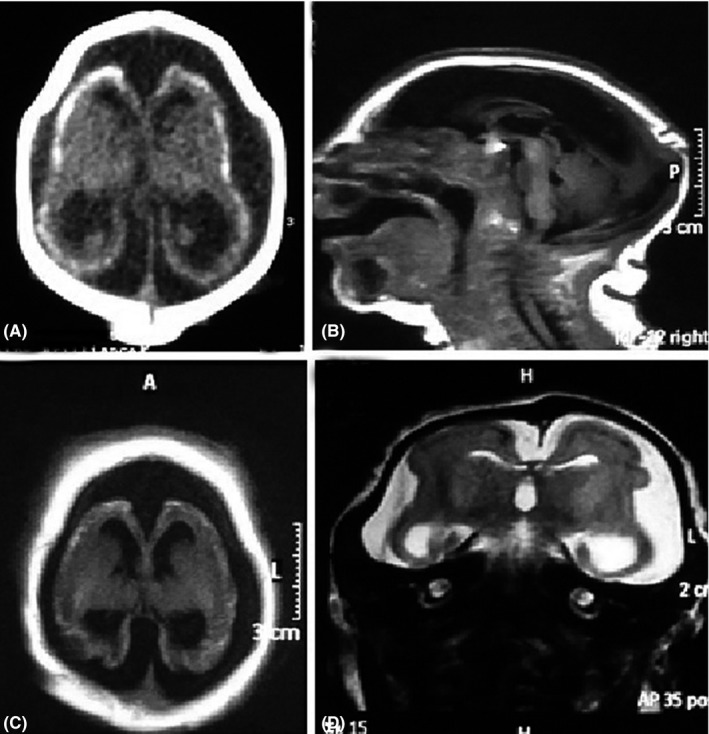
Severe microcephaly, cerebral atrophy with ventriculomegaly, and prominent cerebrospinal fluid space. Axial CT scan (A) shows extensive, punctate cortico‐subcortical calcifications, cerebral atrophy, and bone overlap. Sagittal T1 MR image (B) shows craniofacial disproportion, occipital protuberance with redundant posterior skinfolds, and a hypoplastic corpus callosum. The fossa is relatively preserved. Axial T1 (C) and coronal T2 (D) demonstrate the abnormal gyral pattern with diffuse undersulcated cortex

Computed tomography (CT) and brain magnetic resonance imaging (MRI) identified microcephaly, cerebral parenchymal atrophy, and ventriculomegaly with an increased subarachnoid space. Diffuse cortico‐subcortical calcifications were observed. There was a simplified gyral pattern, lissencephaly, and a hypoplastic corpus callosum. Craniofacial disproportion, exuberant external occipital protuberance, and posterior skin folds were observed. The brainstem and cerebellum were normal (Figure 1).

At 4 months of age, the child attended a rehabilitation program at Sarah Hospital. The child had serologic tests at this age and results were positive for dengue IgG, negative for dengue IgM, positive IgG for herpes I/II, and negative for toxoplasmosis, syphilis, rubella, and cytomegalovirus.

The karyotype investigation of blood cells showed mosaicism 47,XY,i(Xq)[7]/46,XY[43]. Parents’ karyotype was normal.

The child had epilepsy, severe microcephaly with normal scalp hair pattern, dysphagia with gastrostomy, and severe neurological impairment. The child died at 5 years old.

## 3 DISCUSSION

Most of the ZIKV patterns of microcephaly are consistent with fetal brain disruption sequence (FBDS), and the spectrum of brain anomalies is more profound and more common than that observed in other congenital infections. Congenital cytomegalovirus can produce a similar phenotype, although the location of the intracranial calcifications may differ.^3^, ^4^ The symptoms of the distinctive phenotype of congenital Zika syndrome are severe microcephaly with a collapsed skull and prominent scalp rugae, profound craniofacial disproportion, exuberant external occipital protuberance, brain calcifications in the junction between cortical and subcortical white matter, and malformations of cortical development, often with a simplified gyral pattern and predominance of pachygyria or polymicrogyria in the frontal lobes. Additional findings are enlarged cisterna magna, abnormalities of corpus callosum (hypoplasia or hypogenesis), ventriculomegaly, delayed myelination, and hypoplasia of the cerebellum and the brainstem.^3^, ^4^

Fetal brain disruption sequence (FBDS) can be caused by infections, mechanical and vascular factors, exposure to toxins and hyperthermia. Because of the normal scalp hair pattern, it is thought that this phenotype is due to progressive brain destruction during the second or third trimester of pregnancy.^3^, ^4^

Fetal brain arrest was the term used to differentiate rare familial cases with similar signs of fetal brain disruption sequence. Most of the patients do not have brain calcifications. Additionally, the pattern and location of calcifications are quite distinct from that seen in congenital ZIKV. In fetal brain arrest, the described cases with calcifications showed subependymal or band like calcifications.^5^

It was observed that a boy was born in Brazil in December 2011 with epidemiological, clinical, and radiologic signs of Zika virus embryopathy. The patient had severe microcephaly, rugated scalp, prominent occipital bone, overlapping sutures, and a normal scalp hair pattern. Brain imaging revealed severe volumetric encephalic reduction with a simplified gyral pattern, ectasia of lateral ventricles and extensive calcification on cortical brain region.

The fact that the child had chromosomal abnormality mosaicism 47,XY,i(Xq)/46,XY does not explain the signs of cerebral damage, and there is no association between microcephaly and mosaicism with isochromosome X abnormalities. The male patients with those chromosomal abnormalities usually show Klinefelter syndrome variant phenotype.^6^

The patient's mother had signs of dengue infection including a fever, rash, and positive dengue serologic tests on gestation. The child also had positive dengue serology IgG at 4 months of age. Cross‐reactivity with dengue virus specific IgG is a possibility.

It was difficult to determine retrospectively whether the mother and the child had been infected by ZIKV. In the case‐control study of,^7^ only 11% of the microcephaly cases tested positive for Zika virus‐specific IgM in serum. The timeframe for detection of ZIKV specific IgM and IgG is uncertain. The sensitivity and specificity of these tests are not known, but they seem to be low.^7^, ^8^

Diagnosis of ZIKV infection in Brazil has been complicated by limitations in laboratory assays, cross‐reactivity between other flavivirus antibodies and by the fact that dengue has been endemic for more than 30 years. Co‐infection with chikungunya virus has also occurred. By September 2014, CHKV epidemics were confirmed in Feira de Santana, Northeast Brazil.^9^

Many studies have examined the correlation between DENV infection and effects during pregnancy. Dengue infection during pregnancy is associated with low birth weight, prematurity, and fetal loss, but there is not enough evidence to prove that the dengue virus could cause teratogenic effects.^10^

Even considering the remarkable resemblance between ZIKV with DENV and West Nile virus, there was no evidence indicating that dengue infection is related to fetal brain disruption.

The putative association between the Zika virus epidemic and concomitant increase in the number of babies born with microcephaly is based on epidemiologic, clinical, laboratory, and experimental studies. Evidence strongly supports a link between ZIKV infection and observed neurological complications.^11^, ^12^

A new challenge has arisen in Brazil with the emergence of ZIKV and co‐circulation with other arboviruses (i.e., Dengue [DENV] and chikungunya virus [CHIKV]). It is likely that the rapid spread of ZIKV in Brazil and concomitant flavivirus infections have a high potential risk of teratogenicity.

According to the phylogenetic and molecular results of Faria et al^2^ in 2016, the estimated introduction of Zika to Americas occurred between May and December 2013, more than 1 year before the virus was first reported.^2^

Naccache et al^13^ in 2016 suggested that Zika virus was present in Salvador‐Bahia in mid‐2014 and was likely introduced from other regions of Brazil rather from outside the country.^13^

The present case strongly suggests embryo‐fetal brain disruptive sequence by the ZIKV. It is possible that the ZIKV could have been circulating in northeast Brazil since 2011, at least 4 years before the epidemic erupted and before the outbreak in Polynesia.

## CONFLICT OF INTEREST

None declared.

## AUTHOR CONTRIBUTIONS

All authors contributed to the preparation of this manuscript. KEFAC, GLCCS: responsible for clinical management of the patient and obtention of clinical data; participated in writing and revision of the manuscript. SFP, ALC, IVB: participated in writing and revision of the manuscript. CMP: performed the cytogenetic analysis; participated in writing and revision of the manuscript.
